# Exploring the microbial savanna: predator-prey interactions in the soil

**DOI:** 10.1038/s44320-024-00033-w

**Published:** 2024-04-08

**Authors:** Laura Sanchis Pla, Jordi van Gestel

**Affiliations:** Developmental Biology Unit, European Molecular Biology Laboratory, 69117 Heidelberg, Germany

**Keywords:** Evolution & Ecology, Microbiology, Virology & Host Pathogen Interaction

## Abstract

Soil ecosystems consist of complex multi-trophic communities of predator and prey microbial species. This Comment proposes that integrative approaches are powerful for understanding predator-prey interactions and for revealing how these interactions shape population dynamics in soil communities.

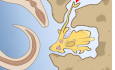

## Teaser

Soils host complex multi-trophic communities with diverse, mostly microbial, predator and prey species, including numerous bacterivorous protists and bacterial prey. The molecular mechanisms underlying microbial predator-prey interactions have thus far mainly been explored using reductionist methods, outside the soil environment and independent from the broader life history strategies that microbes display in soils. In this Comment, we advocate for an integrative research approach, combining molecular systems biology and microbial ecology, to investigate how predator-prey interactions shape microbial life history strategies and thereby population dynamics in natural soil communities.

“If I could do it all over again, and relive my vision in the 21st century, I would be a microbial ecologist. Ten billion bacteria live in a gram of ordinary soil, a mere pinch held between thumb and forefinger. They represent thousands of species, almost none of which are known to science. Into that world, I would go with the aid of modern microscopy and molecular analysis. I would cut my way through clonal forests sprawled across grains of sand, travel in an imagined submarine through drops of water proportionately the size of lakes, and track predators and prey in order to discover new life ways and alien food webs.” (Wilson, 1994).

In his autobiography, the naturalist E. O. Wilson eloquently described the foreign ecology of soils, which despite their importance, remain among the most elusive ecosystems on the planet: soil communities are incredibly diverse; strongly structured in both time and space and largely hidden from our sight, making it practically impossible to directly observe how microbes are interacting. Over the last decade, many studies have used sequencing-based approaches to map out soil diversity, revealing communities that are largely centered around bacterial and fungal growth. Soil communities also contain legions of predators, including protists, nematodes, springtails, and mites (Potapov et al, 2022). These predators differ in size, activity, locomotion, and diet, and engage in many unexpected interactions, from nematode-trapping fungi to pack-hunting amoebae (Thakur and Geisen, 2019). Together with their prey, predators give rise to a ‘microbial savanna’, a complex food web that drives belowground community dynamics.

Bacterivorous protists are among the most diverse predators in the soil. Being higher up in the food chain, these protists are far less abundant than their prey, but their impact on bacterial populations can nonetheless be enormous. A single gram of soil easily contains tens of thousands of bacterivorous protists, each one of them can consume hundreds to thousands of bacterial cells per hour, and protistan populations can grow rapidly with many protists dividing every few hours under favorable conditions. Such strong predation pressures limit bacterial growth and alter the community composition, while conversely, changes in bacterial densities directly affect protistan growth, linking population dynamics in both predator and prey (Thakur and Geisen, 2019).

**Figure 1:**
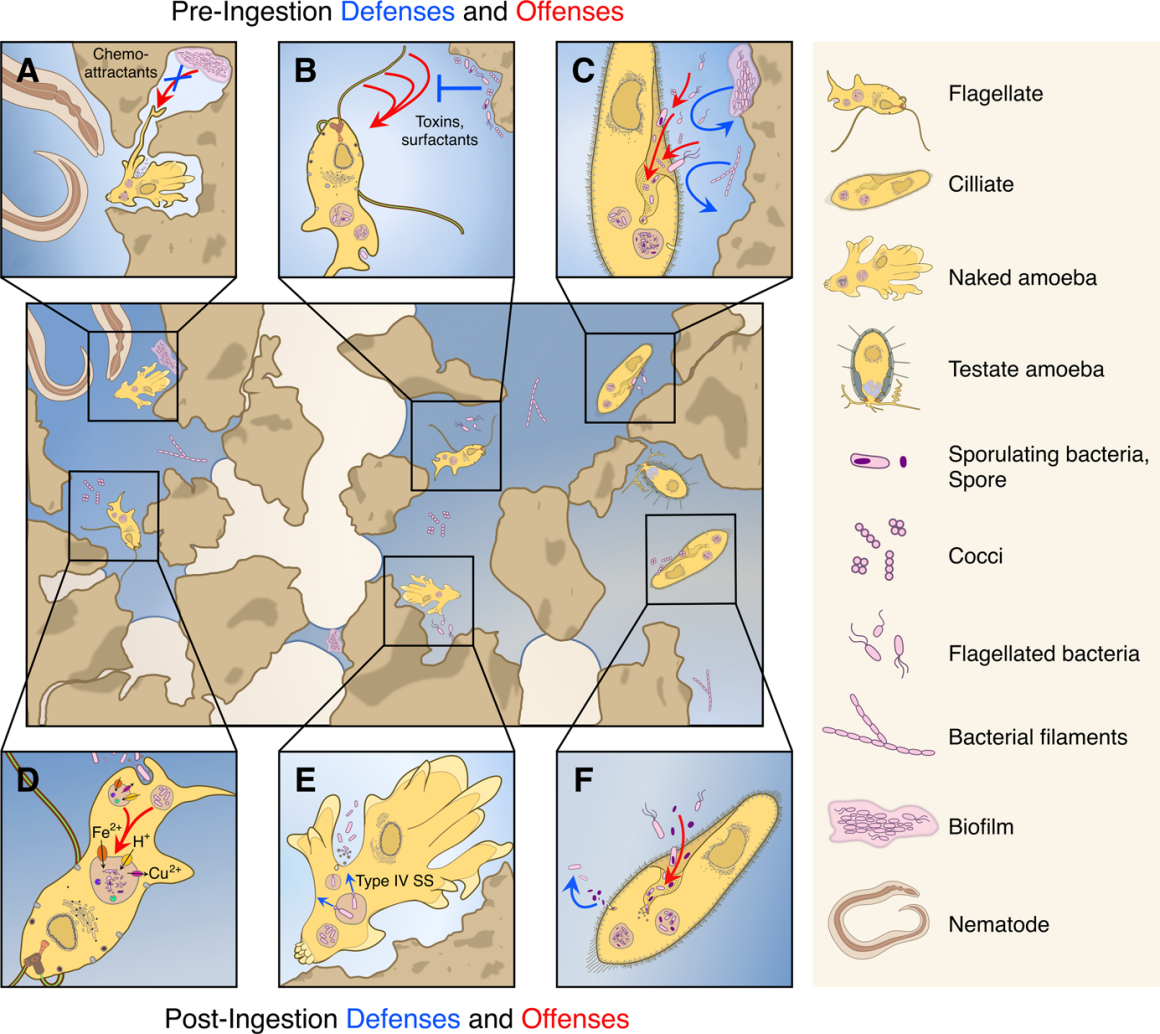
Offense and defense mechanisms in the soil’s microbial savanna. The interplay between protistan predators and bacterial prey within the soil environment is reflected in their diverse lifestyles. (**A**) An ameba can stretch its thin pseudopodia between micropores to reach its bacterial prey, attracted by chemotactic cues produced by the microbial community. However, bacteria can protect themselves by masking or minimizing the production of such cues. Additionally, being agile predators, naked amoebae are themselves exposed to other predators, such as nematodes. (**B**) A flagellated protist uses its flagellum to detach bacterial cells from the soil’s surface. But these can, in turn, express toxins or surfactants to drive the predator away. (**C**) The cilia of protists can create water currents that direct bacteria towards their cytopharynx, an invagination acting as a “mouth”, promoting their ingestion. However, bacterial differences in size and shape, like filamentous bacteria or biofilms, can withstand the current or outsize the cytopharynx, thereby preventing ingestion. (**D**) After ingestion and during phagolysosome maturation, bacteria are exposed to a series of stressors, including digestive enzymes, toxic metals, and high acidity. (**E**) While non-pathogenic bacteria are often easily digested during phagocytosis, intracellular pathogens, such as *L. pneumophila*, can secrete effector proteins through their type IV secretion system and replicate within the phagosome. (**F**) Spores can passively resist digestion within the phagosome. Figure inspired by Erktan et al, 2020 and with modified protist figures from Keeling and Eglit, 2023.

In search of their prey, protists employ diverse offense mechanisms that promote the detection, ingestion, and digestion of bacterial cells, as apparent from their diverse feeding strategies (Esteban and Fenchel, 2020). Raptorial protists, for example, actively move towards their prey, like amoebae that can sense minute changes in chemotactic cues and capture prey using their pseudopodia (Fig. 1A). Other protists, including many ciliates and dinoflagellates, are filter feeders, ingesting large volumes of water to capture prey species. These predators can either be sessile—attached to soil particles—or planktonic—suspended in water-filled pores—and frequently express specialized organelles or cell extensions to capture and kill their prey (Leander, 2020) (Fig. 1B,C). With the expanding number of omics approaches, our understanding of the molecular mechanisms underlying these diverse feeding strategies is advancing rapidly.

After ingestion, protists digest their prey through a common process of phagocytosis, which is at the heart of their predatory lifestyle. During phagocytosis, bacteria are exposed to a series of stressors that mediate their digestion, including acidification, enzymatic digestion, oxidative stress, metal deprivation, and metal poisoning (Fig. 1D). The cocktail of enzymes (e.g., hydrolases and proteases), as well as their effectiveness in digesting different bacterial species, varies between protists: some bacteria are more resilient to digestion than others, thereby increasing the time and energy needed for their consumption. One would therefore expect that the stressors expressed during phagocytosis are tailored to digest the bacterial species that each predator is likely to encounter in the soil.

The diversity of offense mechanisms in protists is mirrored by diverse defenses in their bacterial prey (Jousset, 2012 and references herein). Similarly to offenses, defense mechanisms can coarsely be divided between pre- and post-ingestion mechanisms. Bacteria can, for instance, secrete small molecules that kill or repel protistan predators, like toxins, reactive oxygen species, and biosurfactants (Fig. 1B). Based on the number of biosynthetic gene clusters, bacteria can, in principle, secrete a vast potential of secondary metabolites. However, the ecological function of most of these secondary metabolites remains unknown. Bacteria can also prevent ingestion by outrunning or outsizing predators: bacterial cells can swim away, become filamentous, and form small aggregates or adhesive surface-bound biofilms (Fig. 1C). In other cases, bacteria might evade detection or lower detection rates by masking their cell envelope through modified lipopolysaccharides.

Even after ingestion, bacteria can express defenses, which either actively or passively prevent digestion. Pathogenic bacteria can, for example, interfere with the phagocytic process by hijacking the phagosome for replication, as seen in *Legionella pneumophila* that modulates its vacuole by secreting effector proteins into the protistan host using the type IV secretion system, akin to the development of *Legionella*-containing vacuoles in human phagocytes (Fig. 1E). Opportunistic pathogens like *L. pneumophila* can resist predation by many protistan species (Park et al, 2020). As such, protistan predation is often thought to promote the emergence of bacterial virulence in humans. But not all defenses actively interfere with phagocytosis. Passive resistance occurs when bacteria resist phagocytic stressors without manipulating the host (Jousset, 2012). *Bacillus subtilis* spores can, for example, resist phagocytosis by *Tetrahymena thermophila* through the formation of an impenetrable spore coat (Fig. 1F).

Given the multitude of offense and defense mechanisms, it is not surprising that offenses in protists and defenses in bacteria are often combined. Bacteria can, for example, simultaneously produce biofilm, form filaments, and secrete biosurfactants. The combination of defenses determines the effective predation rate of protistan predators. To coordinate expression, defenses are often co-regulated in response to, for example, quorum-sensing signals or environmental cues. Similarly, protists undergo strong gene expression changes in response to the available prey species and can modulate their offenses, like the digestive enzymes expressed during phagocytosis. Although response mechanisms are widespread in both predator and prey, their molecular underpinnings, as well as ecological impact, often remain unknown. How do bacteria assess the predation risk in their environment and, conversely, how do protists optimize their feeding strategy in response to the available prey species?

There is a limit to the number of offense or defense mechanisms microbes can express. Many mechanisms are costly and affect important life history traits, like growth, dispersal, and survival. Biofilms, for instance, protect bacteria against predation, but can simultaneously lower growth rates and limit dispersal, by trapping cells in adhesive extracellular polysaccharides. By reducing the predation risk through biofilm formation, bacteria might thus limit their ability to find new resources. Similarly, the time and energy protists invest in digesting bacterial prey might strongly differ between prey species. Protists might therefore benefit from consuming more digestible prey species first, depleting those species from the community. Feeding strategies can also directly affect the survival chances of protists themselves. For example, the agility of amoebae makes it possible to engulf prey in micron-scale pores, but can also make them susceptible to predation by larger predators, like nematodes (Fig. 1C). Testate amoebae reduce this risk by hiding in a protective shell. Understanding life history trade-offs is central to understanding the repertoire of offense or defense mechanisms microbes express.

The life history strategies of protistan predators and bacterial prey can only be fully understood in the context of the soil environment, whose strong spatial structure inevitably affects the spatial and temporal scale at which microbes interact (Erktan et al, 2020). Ecology should therefore be leading in studying predator–prey interactions. This poses a major challenge because microbes are difficult to monitor directly in heterogeneous soil environments. In addition, to quantify how offense and defense mechanisms affect life history strategies, we need to perturb them and determine their effect on growth, dispersal, and survival. Only in this way, can we understand how life history trade-offs shape the repertoire of expressed mechanisms. We think this calls for an integrative research approach, combining methods from molecular systems biology and microbial ecology, which starts from the offense and defense mechanisms predator and prey species express, and systematically studies their impact on microbial life history strategies and community dynamics in soils.

Recent technological advances strongly promote such an integrative research approach. Advances in gene engineering, like CRISPR, make it possible to create large-scale mutant libraries in an increasing number of species, thereby paving the way to perturb offense and defense mechanisms in a high-throughput fashion (Stewart et al, 2022). In addition, advances in parallel culturing make it possible to study microbial interactions across hundreds of culturing conditions and community compositions. These approaches are complemented by microfluidic experiments with synthetic soils, where we can directly monitor how microbes interact at a spatial and temporal scale that mimics that of a soil environment (Erktan et al, 2020). Such experiments can offer key insights into spatiotemporal community dynamics affecting microbial life history strategies. To date, many of the above methods are applied to either bacteria or protists in isolation, but in the future, we think these efforts could easily be expanded to study the predator–prey interactions that dominate natural soil environments.

By connecting approaches across fields, from molecular systems biology to microbial ecology, we can investigate how life history strategies of protistan predators and bacterial prey arise and evolve. These strategies ultimately shape microbial communities and, thereby, the belowground microbial savanna; a savanna filled with “new life ways and alien food webs” (Wilson, 1994) that await to be discovered.
